# Effect of Aidi Injection plus TACE on Hepatocellular Carcinoma: A Meta-Analysis of Randomized Controlled Trials

**DOI:** 10.1155/2018/9196409

**Published:** 2018-12-17

**Authors:** Yaoyao Dai, Sicheng Gao, Xing Liu, Qin Gao, Lan Zhang, Xingliang Fan, Junfeng Zhu

**Affiliations:** ^1^Department of Hepatology, Shanghai Municipal Hospital of Traditional Chinese Medicine, Shanghai University of Traditional Chinese Medicine, Shanghai 200070, China; ^2^Department of Central Laboratory Medicine, Shanghai Municipal Hospital of Traditional Chinese Medicine, Shanghai University of Traditional Chinese Medicine, Shanghai 200071, China; ^3^Department of Graduate School, Shanghai University of Traditional Chinese Medicine, Shanghai 201203, China

## Abstract

We aim to conduct a meta-analysis of studies on the effect of Aidi injection combined with TACE in the treatment of hepatocellular carcinoma (HCC). China National Knowledge Infrastructure (CNKI), Wanfang Database, Chinese Biomedical Literature Database (CBM), Chinese Science and Technology Periodical Database (VIP), Allied and Complementary Medicine Database (AMED), EMBASE, Web of Science, PubMed, and Cochrane Library databases to October 1, 2017, were searched to collect the studies. The data analysis was performed using RevMan 5.3 software. Totally 20 clinical trials with 774 (the experimental group: 447 cases; the control group: 327 cases) HCC patients were finally included in this meta-analysis. Meta-analysis results showed that Aidi injection combined with TACE can, to some extent, enhance the clinical effect and improve the overall survival. Meanwhile, it can increase HCC patients' quality of life. Additionally, Aidi injection plus TACE can reduce adverse events including leukopenia, gastrointestinal reaction, and liver damage in HCC patients (all P < 0.05). Therefore, Aidi injection plus TACE may significantly enhance the clinical effect, suggesting that the combination of TCM and western medicine is promising. The exact outcome needs rigorously designed performances, multicenter, and large randomized controlled trials.

## 1. Introduction

Hepatocellular carcinoma (HCC) is one of the most common malignant tumors in the digestive system [1]. Most HCC patients were diagnosed in an advanced stage but have lost the chance of operation. Hepatectomy was the only suitable operation in the early stage. More than 70% of tumors were found to be in an advanced stage [2]. Transcatheter arterial chemoembolization (TACE) is the main treatment for unresectable hepatocellular carcinoma. However, the long-term efficacy of this treatment is not ideal, and it often inhibits the immunity of the organism, aggravating the impairment of liver function and reducing the quality of life in the control and removal of tumor. Therefore, finding a way to reduce liver damage and improving the clinical efficacy and quality of life have become the key issue. Aidi injection is mainly composed of Cantharidin, Astragalus extract, and Acanthopanax senticosus, Chinese traditional medicine injections [3]. In recent years, Aidi injection combined with TACE has been widely used in the treatment of unresectable HCC. However, the results of these clinical trials are not completely consistent, and there is no accurate and scientific evaluation of the efficacy of combined therapy. For further exploring the role of Addie injection combined with TACE in the treatment of HCC, we systematically evaluated 20 related clinical trials.

## 2. Materials and Methods

### 2.1. Inclusion Criteria

For “Design” type, they are RCTs using Aidi injection combined with TACE for HCC patients.

For participants, clinical diagnosis must meet the diagnostic standard by pathology, cytology, or image inspection. The group of trials added that Aidi injection apart from the TACE that was used by the group of control. We had not set any restrictions on gender, race, and literary language. The outcome should include one or more indices as follows: clinical curative efficiency, overall survival, KPS score evaluation, and adverse events.

### 2.2. Exclusion Criteria

Patients were not diagnosed with hepatocellular carcinoma. The experiment was not a randomized controlled trial. Interventions were not the comparison between Aidi injection combined with TACE and TACE alone in the treatment of HCCs. The study was a review, a commentary, an animals' experiment, a case observation, a duplicated literature, and a non-injection formulae literature.

### 2.3. Research Strategy and Information Sources

We have searched China National Knowledge Infrastructure (CNKI), Wanfang Database, Chinese Biomedical Literature Database (CBM), Chinese Science, Technology Periodical Database (VIP), Allied and Complementary Medicine Database (AMED), EMBASE, Web of Science, PubMed, and Cochrane Library databases, with no language restrictions and with retrieval deadlines to October 1st, 2017. The following medical subject headings were used: “hepatocellular carcinoma”; “primary liver cancer”; “Traditional Chinese Medicine”; “Aidi injection”; “TACE”; and electronic searches were supplemented with manual searches of reference lists used in all of the retrieved review articles, primary studies, and meetings abstracts to identify other studies which were not found in the electronic searches. Using Excel to formulate data extraction table, the two researchers (DYY and LX) independently read the literature and abstract, screening out the relevant literature, reviews, and pharmacological experiments, such as the test for control, by reading the full text to determine whether it meets the inclusion criteria. Data extraction and quality assessment were also independently performed by the two researchers. In case of disagreement, it solved through discussion or decision by the third party. The lack of information was supplemented by contact with the authors in charge of the clinical trials.

### 2.4. Definitions

The diagnosis of hepatocellular carcinoma should be based on guidelines: the clinical curative efficiency according to the World Health Organization (WHO) standards [4], complete response (CR), partial response (PR), no change (NC), progressive disease (PD), the total effective rate = (number of CR cases + PR cases)/total number of cases × 100%; KPS score: according to the Karnofsky Performance Score grading system, the fact that KPS increased 10 points after the treatment indicated improved patients' quality of life. On the contrary, the fact that KPS decreased 10 points after the treatment indicated reduced patients' quality of life.

### 2.5. Statistical Analysis

Cochrane RevMan 5.2 was used for meta-analysis. Categorical variables using relative risk (relative risk, RR) for the analysis of curative effect statistics and continuous variables using mean difference (mean, difference, MD) were both calculated through 95% confidence interval (confidence interval, CI). Chi-square test was used to analyze the statistical heterogeneity. I2 was used to evaluate the heterogeneity inconsistency: I2≤25% for low heterogeneity, 25%-50% for moderate heterogeneity, more than 50% for the high degree of heterogeneity. No statistical heterogeneity was studied by fixed-effect model combining with analysis. If the case results have significant heterogeneity, a random-effect model is used. Test results are listed in forest maps or tables, and publication bias is shown by the symmetry of funnel plots.

## 3. Results

### 3.1. Characteristics of Included Studies

**Figure 1:**
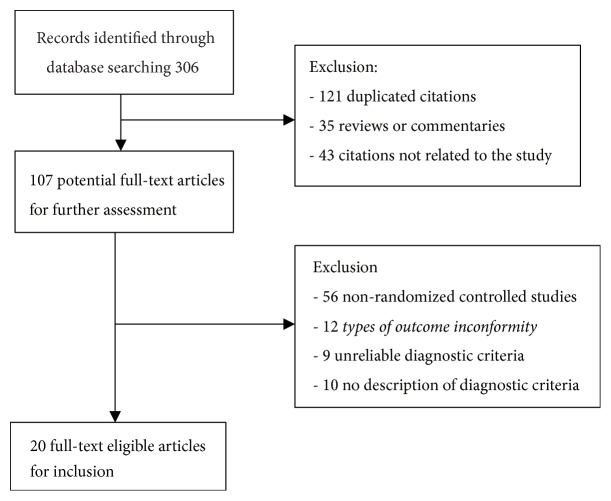
Flow chart of study selections.

We identified 306 potentially eligible trials from electronic database researches. Among these articles, 20 clinical trials [1, 5–13] with 774 (the experimental group: 447 cases; the control group: 327 cases) hepatocellular carcinoma patients were finally included in this meta-analysis. The study selection was shown in Figure 1. The general characteristics of the included studies are shown in Table 1.

### 3.2. Methodological Quality Assessment

**Figure 2:**
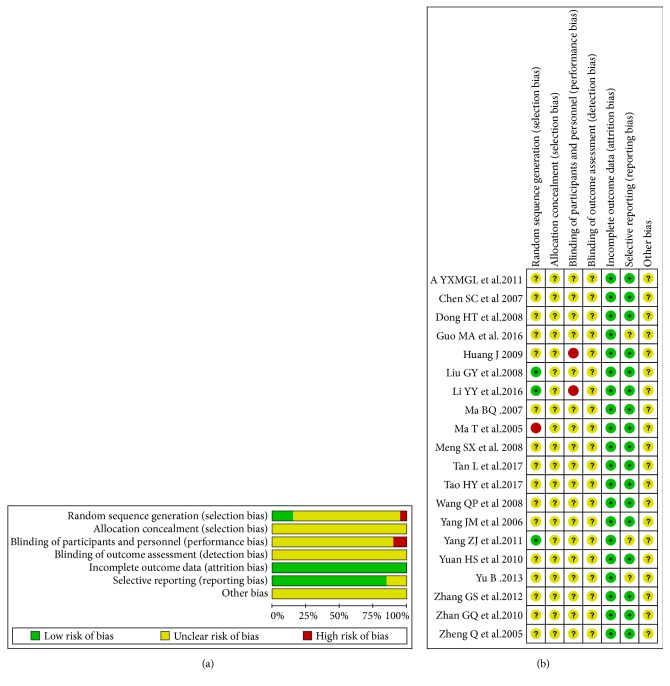
Risks of bias graph (a) and risks of bias summary (b).

Using Cochran system evaluation method, evaluation of random sequence generation, allocation concealment, blinding of participants and personnel, blinding of outcome assessment, incomplete outcome data, selective reports, and other bias in the studies were conducted. the outcomes were expressed as “low risks,” “high risks,” and “unclarity.” Among the 20 experiments, 4 experiments described the random allocation method. All the included studies were not described as blind methods. Therefore, it counted that there were selective bias and implementation bias. Other bias types were not clear. Characteristics and quality of all included studies are shown in Figure 2.

### 3.3. Clinical Curative Efficiency

**Figure 3:**
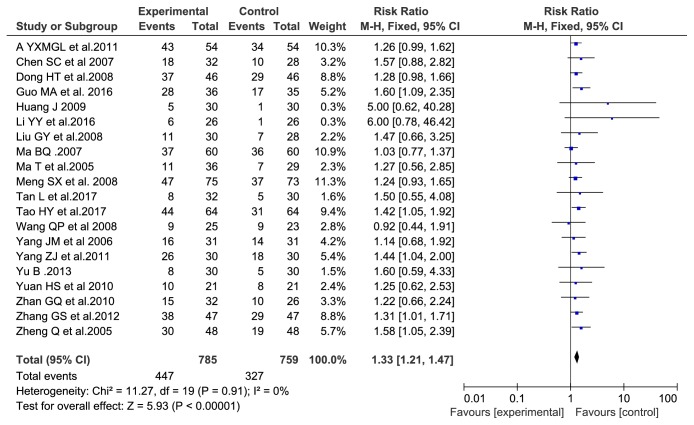
Clinical curative efficiency.

We identified twenty trials [1, 5–13] with 774 participants and evaluated the clinical curative efficiency. There was no heterogeneity between the trials (P = 0.91, I2 = 0%) and a fixed-effects model used (RR, 1.33; 95% CI: (1.21, 1.47), P < 0.00001), which indicated that there was a statistically significant difference between groups of Aidi injection plus TACE and TACE alone which indicated that Aidi injection plus TACE in the treatment was better than TACE alone. The results are shown in Figure 3.

### 3.4. Overall Survival

**Figure 4:**
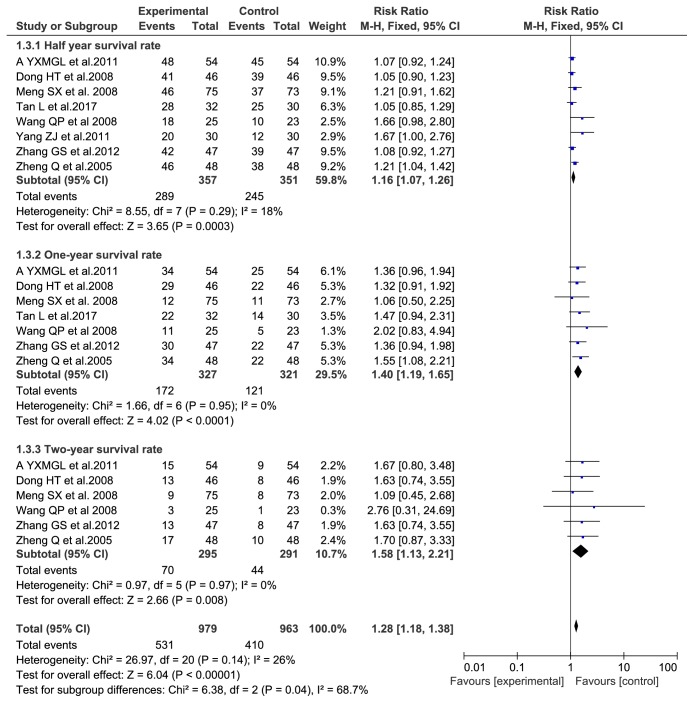
Overall surviving comparisons.

Half-year survival rates were evaluated in eight trials [1, 5, 7, 12, 13] with 534 participants. No heterogeneity was found among the included trials (P = 0.29, I2 = 18%). Fixed-effects model (RR = 1.16, 95% CI: (1.07, 1.26), P = 0.0003) was used for meta-analysis. One-year survival rates were evaluated in seven trials [1, 5, 7, 12, 13] with 534 participants. Two-year survival rates were evaluated in six trials [1, 5, 7, 12, 13] with 293 participants. No heterogeneity among the included trials (P = 0.95, I2 = 0% and P = 0.97, I2 = 0%) using fixed-effects model, one-year survival rates (RR = 1.40, 95% CI: (1.19-1.65), P < 0.0001), and two-year survival rates (RR =1.58, 95% CI: (1.13-2.21), P = 0.008). The half-year survival rates, one-year survival rates, and two-year survival rates of TACE combined with Aidi injection as an experimental group were significantly higher than those of a control group treated with TACE alone; the results are shown in Figure 4.

### 3.5. KPS Score Evaluation

**Figure 5:**
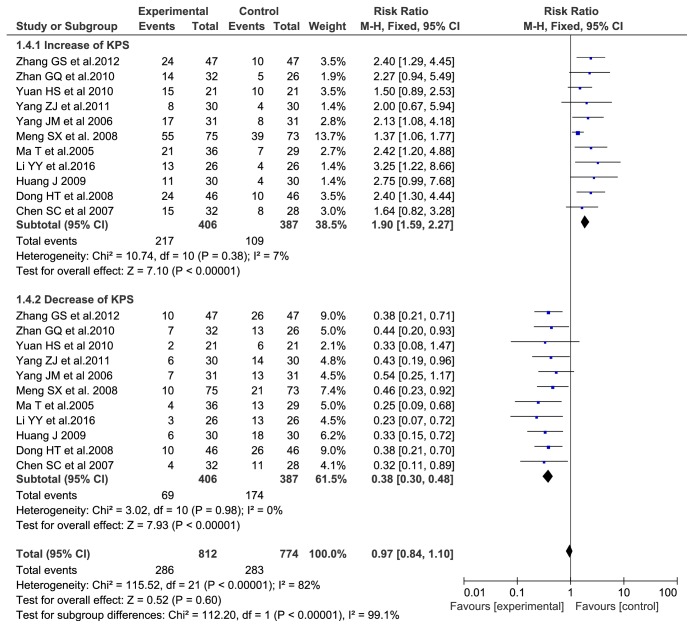
KPS score evaluation.

We identified eleven trials [1, 5, 7–9, 11, 12, 14] including 566 participants with the outcome measurement of KPS score. The result showed that there was no statistical heterogeneity among studies: KPS score increased rates (P = 0.38, I2 = 7%) and KPS score decreased rates (P = 0.98, I2 = 0%), which used the fixed-effects model. The results indicated that the experimental group can significantly improve the quality of life of patients compared with the control group (RR = 1.90, 95% CI: (1.59, 2.27), P < 0.00001). Moreover, the descending rate of KPS was lower in the experimental group than that in the control group (RR = 0.38, 95% CI: (0.30, 0.48), P < 0.00001). So Aidi injection plus TACE can improve quality of life when compared with TACE alone. The results are shown in Figure 5.

### 3.6. Adverse Events

**Figure 6:**
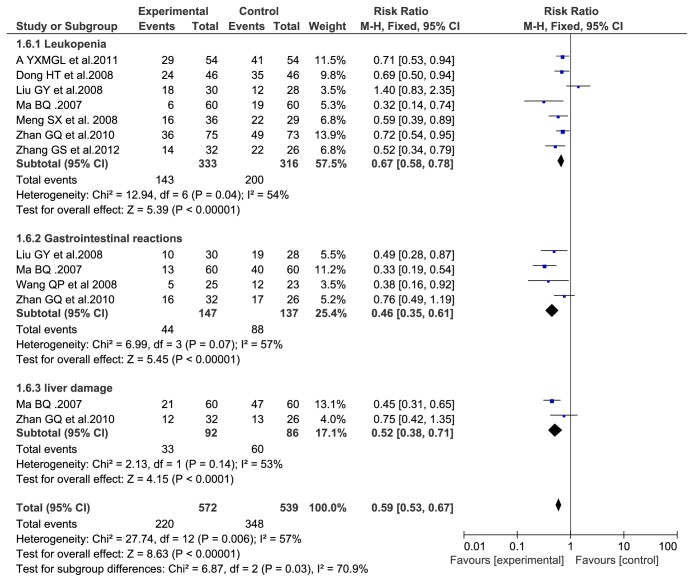
Adverse events.

The common side effects of TACE are bone marrow suppression phenomenon, such as the decline of platelet leukocyte, etc.; gastrointestinal symptoms such as abdominal pain, nausea, and vomiting; other adverse reactions including abnormal liver function (mainly transaminase elevations), but they were mild and could be alleviated after symptomatic treatment. 7 studies [5–7, 21, 22] reported adverse effects of TACE combined with Aidi injection versus TACE alone in the treatment of HCC. Leukopenia, gastrointestinal reaction, and liver damage were obvious heterogeneity (I2 = 54%, I2= 57% and I2 = 53%, resp.), by the random effects model analysis, leukopenia (RR = 0.67, 95% CI: (0.58, 0.78), P < 0.00001), gastrointestinal reaction: (RR = 0.46, 95% CI: (0.35, 0.61), P < 0.00001), and liver damage (RR = 0.52, 95% CI: (0.38, 0.71), P < 0.0001); the results suggested that with TACE combined with Addie injection in the treatment of primary liver cancer leukopenia, gastrointestinal reaction, and liver damage occurrence rate was lower than the TACE alone; the difference was statistically significant. The results are shown in Figure 6.

### 3.7. Publication BIAS

**Figure 7:**
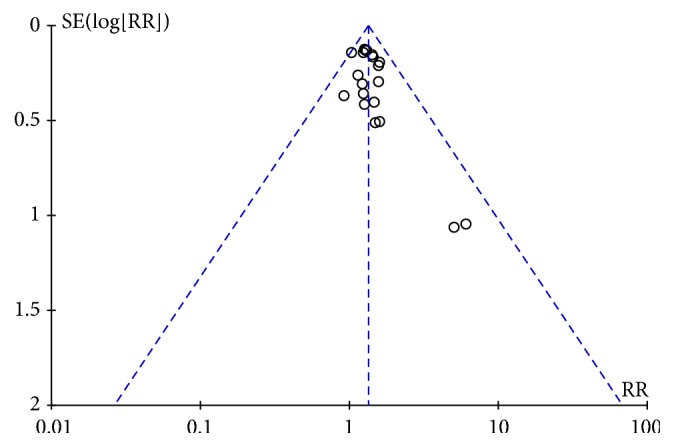
The funnel plots based on the data of the overall efficacy.

Cochrane RevMan 5.2 was used to draw the funnel plot. The plot was asymmetric (Figure 7), suggesting that the publication bias may occur in this study.

## 4. Discussion

The most effective treatment for hepatocellular carcinoma is surgical resection and liver transplantation. However, there is an opportunity for surgical resection of about 20%-30% in patients [23]. Liver transplantation is expensive; TACE is currently recognized as one of the most common methods of nonsurgical treatment of hepatocellular carcinoma, but due to adverse reactions and traumatic treatment after TACE, it often leads to postembolization syndrome. The main manifestations were fever, pain, nausea, and vomiting. In addition, other adverse reactions can also be seen, such as puncture bleeding, leukopenia, transient liver dysfunction, renal dysfunction, and difficulty urinating. These adverse reactions, to a certain extent, reduce the quality of life of patients. After TACE, tumor necrosis and hypoxia caused by increased vascular endothelial growth factor (VEGF) promote tumor angiogenesis, resulting in tumor recurrence [24]. Modern research confirmed [15–17] that Aidi injection can inhibit the expression of VEGF protein in tumor tissue to achieve the purpose of inhibiting tumor growth.

Primary liver cancer treatment is mainly inclined to comprehensive treatment; a large number of clinical trials confirmed that the efficacy of traditional Chinese medicine in the field of liver cancer has a significant effect, not only improving the prognosis and quality of life, but also enhancing patient survival rate. Aidi injection is based on the principle of strengthening vital qi to eliminate pathogenic factor in addition to one of the Chinese medicines, as cantharides, ginseng, Astragalus, and Acanthopanax. The main role is “clearing away heat and toxic material,” Xiao yu San jie. Aidi injection has obvious inhibitory effect on solid tumor in mice [25], which can enhance the body's nonspecific and specific immune function, improve the body's stress ability, and associate with anticancer drug 5-Fu. CTX and radiotherapy have synergized action. In vitro tumor inhibition experiments show that [18] the goods on the cancer cells have direct killing and inhibition. Meta-analysis of randomized controlled trials has demonstrated its role in non-small cell lung cancer [26] and gastric cancer [27]. In modern pharmacological studies: Astragalus polysaccharides have significant immunomodulatory activity [19], hepatoprotective and antioxidation effects [20, 28], and antitumor effect [29] which may be related to their ability to enhance the expression of IL-1*α*, IL-2, IL-6, and TNF-*α*, decrease IL-10, and downregulate MDR1 mRNA and P-GP expression levels [30]. Ginsenosides (such as ginsenoside Rg3, Rh2) in various models in tumor cells and vascular endothelial cells show antitumor and antiangiogenic effects [31]. Acanthopanax senticosus saponins also have antitumor and immunomodulatory effects. The study may be related to the activation of macrophages and NK cells [32]. Some researchers suggest that it is related to inhibiting the expression of VEGF and VEGF mRNA [33]. Cantharidin has potent antitumor activity and induces a variety of tumor cell apoptosis [34]. Furthermore, cantharidin can increase the white blood cells and reduce the occurrence of bone marrow suppression [35].

The evaluation system included in this study also has limitations, which will affect the outcome of the argument strength: ① The inclusion studies did not mention the basis for the sample size estimates, the sample size is small, and the design of individual research methods is not high, with no long-term follow-up, which would reduce the validity of evidence. ② All studies did not carry out blind assessment; it may influence the objectivity of the outcome. ③ All trials mentioned allocation concealment, which might bring selective bias. ④ All studies came from China, so publication bias will occur.

By meta-analysis of randomized controlled trials of hepatocellular carcinoma in recent years by TACE combined with Aidi injection, we can conclude that TACE combined with Aidi injection in the treatment of hepatocellular carcinoma may really improve the efficiency of clinical disease, slow down the progress of disease, improve patients survival rate and quality of life, enhance the immunity of patients, and reduce the adverse reactions caused by TACE. Therefore, TACE combined with Aidi injection is superior to TACE alone in the treatment of hepatocellular carcinoma, which provides evidence for clinical decision-making. But the detailed mechanism of how Aidi injection works in TACE is not completely clear so far and the limitations quality and quantity of included studies were relatively inadequate. Thus, it is necessary to carry out more high quality, multicenter, large sample, prospective, randomized, double-blind clinical trials to be further confirmed or conducted real-world research in the future.

## Figures and Tables

**Table 1:** Characteristics of the randomized controlled trials included in this study.

study	Number of cases			Control regimen (TACE)	Aidi intervention	therapeutic courses (days/cycles)	HCC staging	KPS score			Randomized method
	T/C	M/F	Age								
Tan L et al. 2017 [5]	30/32	39/23	20-70	L-OHP+ADM+5-FU	50 ml/d	30 d*∗*2	II, III, IV	NA			NA
Dong HT et al. 2008 [6]	46/46	68/24	28 - 77	HCPT+5-FU	60 ml/d	28 d*∗*2	NA	>60			NA
Liu GY et al. 2011 [7]	30/28	40/18	47	ADM+MMC+5-FU	60 ml/d	15 d*∗*4	I, II, III		*⩾*70分		Random number table
Zhang GS et al. 2012 [8]	47/47	68/26	29-78	HCPT+5-FU	100 ml/d	28 d*∗*2	II, III, IV	>60			NA
Meng SX et al. 2008 [9]	75/73	97/51	55.2	THP+5-FU	50 ml/d	14 d*∗*2	II, III, IV	>60			NA
Ma T et al. 2005 [10]	36/29	5/60	47	DDP+HCPT+5-FU	50 ml/d	10 d*∗*1-2	I, II, III	>60			Medical record card number
Tao HY et al. 2017 [11]	64/64	77/59	40-75	L-OHP+EPI+5-FU	60 ml/d	28 d*∗*4	I, II, III	>50			NA
Yu B. 2013 [12]	30/30	29/31	18-65	DDP+ADM+MMC+5-FU	50 ml/d	60 d*∗*1	NA	>60			NA
A YXMGL et al. 2011 [13]	54/54	80/28	28-77	HCPT+5-FU	60 ml/d	21 d*∗*2	II, III, IV	>60			NA
Guo MA et al. 2016 [14]	36/35	47/24	55.6	ADM+MMC+5-FU	60 ml/d	14 d*∗*2	NA	NA			NA
Yang JM et al 2006 [15]	31/31	50/12	27 - 68	ADM+DDP+5-FU	50 ml/d	15 d*∗*2	NA	NA			NA
Huang J 2009 [16]	30/30	51/9	45.1	ADM+DDP+5-FU	80 ml/d	15 d*∗*2	NA	30 - 60			NA
Yuan HS et al 2010 [17]	21/21	36/6	28-74	MMC+THP+5-FU	50- 100 ml/d	10 d*∗*1-2	NA	NA			NA
Chen SC et al 2007 [18]	32/28	41/49	36-70	MMC+HU+5-FU	60 ml/d	21 d*∗*2	NA	>60			NA
Li YY et al. 2016 [19]	26/26	32/20	21-80	DDP+EPI+5-FU	40-80 ml/d	15 d*∗*2	III, IV	30 - 60			Random number table
Yang ZJ et al. 2011 [20]	30/30	53/7	26-69	MMC+EPI+HCPT+5-FU	50-100 mL	10 d*∗*3	NA	*⩾*70			Envelope method
Zheng Q et al. 2005 [21]	48/48	73 /25	50.6	THP+HCPT+5-FU	50 ml	28 d*∗*2	NA	>60			NA
Zhan GQ et al. 2010 [22]	32/26	46/12	21-65	MMC+HCPT+EPI+5-FU	50 ml	20 d*∗*2	II, III	>50			NA
Ma BQ. 2007 [23]	60/60	78 /42	17-82	DDP+CF+MMC+5-FU	50 ml	15 d*∗*3	NA	*⩾*60			NA
Wang QP et al 2008 [24]	25/23	37/11	29-68	MMC+ADM+5-FU	100 ml/d	40 d	NA	NA			NA
